# A qualitative study to understand the challenges of conducting randomised controlled trials of complex interventions in metastatic colorectal cancer

**DOI:** 10.1186/s13063-025-08811-z

**Published:** 2025-03-19

**Authors:** Niamh McKigney, Jenny Seligmann, Maureen Twiddy, Simon Bach, Faheez Mohamed, Nicola Fearnhead, Julia M. Brown, Deena P. Harji

**Affiliations:** 1 Clinical Trials Research Unit, Leeds Institute of Clinical Trials Research, University of Leeds, Leeds, UK; 2 Leeds Institute of Medical Research at St. James’s, University of Leeds, St. James’s University Hospital, Leeds, UK; 3 Institute of Clinical and Applied Health Research, Hull York Medical School, University of Hull, Hull, UK; 4 Department of Surgery, University Hospitals Birmingham, Birmingham, UK; 5 Peritoneal Malignancy Institute, Basingstoke and North Hampshire Hospital, Basingstoke, UK; 6 Department of Colorectal Surgery, Cambridge University Hospitals, Cambridge, UK; 7 Department of Colorectal Surgery, Manchester University NHS Foundation Trust, Manchester, UK

**Keywords:** Challenges, Randomised controlled trials, Metastatic colorectal cancer

## Abstract

**Background:**

The use of interventions such as major liver and lung resection, radiofrequency ablation and transarterial chemoembolization in the management of metastatic colorectal cancer (mCRC) is now relatively commonplace in clinical practice. However, the evidence base regarding these treatments is limited with a lack of high-quality data from randomised controlled trials (RCTs). The aim of this study was to understand the challenges associated with conducting RCTs in advanced mCRC and to identify potential strategies to overcome them, with a view to improving trial design and delivery in this setting.

**Methods:**

A qualitative study was undertaken with professionals involved in mCRC trials. Participants were identified using trial registries to identify relevant trials. Individual semi-structured, in-depth qualitative interviews were undertaken online using a topic guide. The principles of thematic content analysis were used for data analysis.

**Results:**

Twelve participants were recruited to the study from six trials; three of the trials had completed, two were either terminated or no longer recruiting and one was open to recruitment. Four major themes were identified, and themes were further subdivided to identify specific challenges and solutions to overcome them. The four themes identified were as follows: trial-related processes, organisational/structural challenges, trial design considerations, and stage IV (metastatic) colorectal cancer-specific factors. Significant challenges were described in relation to funding, ethical approval processes, equipoise, patient preferences, logistical issues in trial delivery, and the advanced nature of mCRC including disease progression and palliative care.

**Conclusions:**

There are a range of strategies which could be implemented to improve the delivery of future trials in this complex setting, from the initial development of a trial through to trial setup, recruitment and follow-up.

**Supplementary Information:**

The online version contains supplementary material available at 10.1186/s13063-025-08811-z.

## Background

Colorectal cancer (CRC) is the third most common malignancy worldwide, with more than 1.9 million new cases in 2020 [1]. Approximately 20% of patients present with distant metastatic disease at the time of index presentation [2], with a further 20–25% developing metastatic colorectal cancer (mCRC) after initial curative treatment [3, 4]. The management of this group of patients is variable; mCRC is a heterogenous disease with many different biological subtypes and has many different oncological treatments, including different timings of treatment delivery. Only a small proportion of patients, approximately 20%, are suitable for curative surgery for both their primary and metastatic disease. The traditional mode of treatment for most patients with mCRC was chemotherapy, however, there has been an expansion in the range of treatment strategies in recent times, including major liver and lung resection, radiofrequency ablation (RFA) and transarterial chemoembolization. This is in part due to the expanding portfolio of oncology-based clinical trials in this setting [5–7]. In contrast, there are a limited number of surgical trials in the mCRC, with much of the evidence base consisting of cohort studies [8–11]. The lack of surgical RCTs addressing the role of major resection, both liver and lung, in mCRC is multifactorial due to the complex treatment strategies, lack of infrastructure and funding, and impractical trial design [12, 13]. This is coupled with issues around surgical equipoise, particularly, when surgical resection is considered to be an ‘effective treatment’ leading to an unwillingness to ‘withhold’ this treatment through randomisation in a trial setting [14]. As a result, there have been several surgical RCTs in the mCRC setting, which have closed early due to a failure to recruit in a timely fashion [15–17]. Despite this, the colorectal community recognise the value and benefit of high-quality research in this setting, with several priority-setting exercises highlighting multiple research questions relevant to mCRC [18–20]. Though the importance of such work is recognised, there remains a lack of evidence regarding the full extent of the challenges to conducting high-quality RCTs in mCRC as understood by triallists undertaking such work. To design and deliver relevant, meaningful, and impactful trials in mCRC requires an in-depth understanding of the current barriers and challenges to conducting high-quality RCTs in this setting, and identifying appropriate solutions and strategies [21]. Identifying challenges to trials in this setting will help guide the design of surgical trials in this setting and will help underpin key research questions in this arena. Therefore, the aim of this study was to gain a better understanding of the challenges and potential solutions to undertaking trials in mCRC through undertaking a qualitative study to give an in-depth understanding from trialists working across mCRC trials.

## Methods

A qualitative study was undertaken in which potential participants were approached following the identification of relevant trials listed on registries. University of Leeds ethical approval was granted for the study. The study is reported in keeping with Standards for Reporting Qualitative Research (SRQR) [22].

### Eligibility criteria and trial identification

The following trial registries were searched to identify mCRC RCTs registered between 2010 – 2023; the National Institutes of Health (NIH) US National Library of Medicine Trials registry (https://clinicaltrials.gov), the International Standard Randomised Controlled Trial Number (ISRCTN) registry (https://www.isrctn.com), the European Union (EU) Clinical Trials Register (https://www.clinicaltrialsregister.eu), the World Health Organisation (WHO) International Clinical Registry Platform (https://trialsearch.who.int), and the Australian New Zealand Clinical Trials Registry (http://www.anzctr.org.au).

RCTs that were ongoing, completed, or discontinued, in adult metastatic colorectal cancer patients, with a surgical intervention in one of the trial arms, were included. Metastatic colorectal cancer was defined as distant solid organ involvement (e.g. liver or lung). Peritoneal metastases were not included; trials in this setting were felt to present unique challenges which would benefit from a separate dedicated study.

### Recruitment

The main contacts of the registered RCTs identified were approached, informed about the study, and provided with an information sheet via email. Contacts listed on publications, including study protocols, related to the study were also approached to participate in the study. Participants were asked to disseminate the information sheet to other members of the trial management group (TMG) with a view to recruiting a variety of professionals involved in metastatic colorectal cancer trials. A purposive sampling strategy was used with the intention to recruit at least four participants for each of the following, though recruitment was not limited to these roles: Chief/Principal Investigators (CI/PI), Trial Managers/Co-ordinators, and Research Nurses. This purposive strategy included the intention to recruit from each of the different categories which were ascribed in relation to the types of trial interventions:

- Surgical resection of the primary tumour,
- Comparing different management strategies for the primary tumour,
- Metastasectomy versus chemotherapy/other therapies,
- Comparing multiple therapies to chemotherapy alone,
- Timing of chemotherapy and liver resection,
- Ablative therapies versus liver resection,
- Comparing surgical approaches to manage liver metastases,
- Comparing surgical approaches to manage lung metastases, and
- Timing of resection for primary cancer versus liver metastases.

Recruitment to the study continued until information power was reached [23], this was assessed iteratively.

### Qualitative interview

Semi-structured, in-depth qualitative interviews were undertaken with each participant using a topic guide (see Additional File 1). Interviews were conducted by NM who is a practicing medical doctor with experience of undertaking qualitative research and delivering research studies in advanced CRC. All interviews were conducted either via telephone or video-teleconference, and audio recorded. Participants provided written consent prior to the interviews.

### Data analysis

All interviews were transcribed verbatim, imported into NVivo 12 and analysed sequentially. The principles of thematic analysis were used to produce a descriptive analysis of the data [24]; transcripts were coded line by line by NM, with each code identifying recurring ideas and concepts. Regular meetings and correspondence with DH were undertaken to discuss the development of codes and themes. The themes were defined and based on the collated data extracts; a detailed analysis was written for each theme. Finally, an account was written to analyse and interpret the data, both within and across themes [24]. The proposed strategies and solutions were identified directly from the interview transcripts, and all were included in the descriptive analysis. Additional solutions were proposed by the research team, which were informed by both their clinical practice and research experience, and are highlighted within the study results.

## Results

A total of 1130 trial records were identified using the search strategy described, all records were reviewed, and 49 trials were identified that met the study eligibility criteria, these are detailed in Additional File 2. Of these 49 trials, 147 individuals were invited to participate in this study. A total of 12 participants working across six trials agreed to participation. All participants who volunteered for the study were included. Four principal investigators, four study coordinators/trial managers, three research nurses and one trial statistician participated in in-depth qualitative interviews. The characteristics of the six trials the participants had worked on are detailed in Table 1. The trials included were run from the UK, Netherlands, Italy, and Japan. Three of the trials included had completed, two were either terminated or no longer recruiting and one was open to recruitment at the time of interview. Of the trials identified, four trials focused on metastatic liver disease, one trial on metastatic pulmonary disease and one trial on multiorgan metastatic disease. The primary endpoint in three trials was a survival outcome i.e. disease-free survival. In two trials, the primary endpoint was a functional outcome, i.e. time to recovery, and the final trial was a feasibility study.

**Table 1:** Characteristics of the trials included

Registry	Trial registry identifier	Country	Trial name	Institute	Year	Sample size (if listed)	Interventions	Primary endpoint	Current status
WHO	JPRN-UMIN 000007787	Japan	EXPERT – Randomised phase III trial of surgery followed by mFOLFOX6 as adjuvant chemotherapy versus peri-operative mFOLFOX6 plus cetuximab for KRAS wild type resectable liver metastases of colorectal cancer	The University of Tokyo	2012	N/A	Pre-operative chemotherapy (mFOLFOX6 plus cetuximab) 6 cycles followed by post-operative chemotherapy (mFOLFOX6 plus cetuximab)	Progression-free survival	Completed
							Surgical hepatectomy followed by chemotherapy (mFOLFOX6)		
WHO	NCT 00874224	Netherlands	ORANGE II – Surgical recovery after left lateral hepatic sectionectomy: laparoscopic versus open surgery	Maastricht University Medical Center	2009	110	Open left lateral hepatic sectionectomy	Time to functional recovery	Completed
							Laparoscopic left lateral hepatic sectionectomy		
ISRCTN	ISRCTN 52040363	UK	LAVA – Liver resection surgery versus thermal Ablation for colorectal liVer metAstases	University of Leeds	2016	330	Thermal ablation of hepatic metastases	2-year disease-free survival	Terminated
							Surgical resection		
Clinical trials	NCT 05138094	Italy	LIVACOR – Minimally invasive LIVer and simultaneous COlorectal Resection	Fondazione Poliambulanza Istituto Ospedaliero	2021	82	Minimally invasive resection of both primary colorectal carcinoma and liver metastases in one procedure	Time to functional recovery	Recruiting
							Minimally invasive resection of the primary colorectal carcinoma and liver metastases in two stages		
ISRCTN	ISRCTN 15067672	Netherlands	ORCHESTRA—A randomised multicentre clinical trial for patients with multi-organ, colorectal cancer metastases comparing the combination of chemotherapy and removing as many visible tumours as possible by surgery or other means versus chemotherapy alone	Radboud University Medical Centre Nijmegen	2020	478	Chemotherapy and maximal tumour debulking including surgery, RFA, transarterial chemoembolization	Overall survival	No longer recruiting
							Chemotherapy		
Clinical trials	NCT 01106261	UK	PulMiCC – A randomised trial of Pulmonary Metastasectomy in Colorectal Cancer	University College London	2012	N/A	Pulmonary metastasectomy	Feasibility of recruitment	Completed
							Active monitoring		

### Themes identified from thematic analysis

**Fig. 1:**
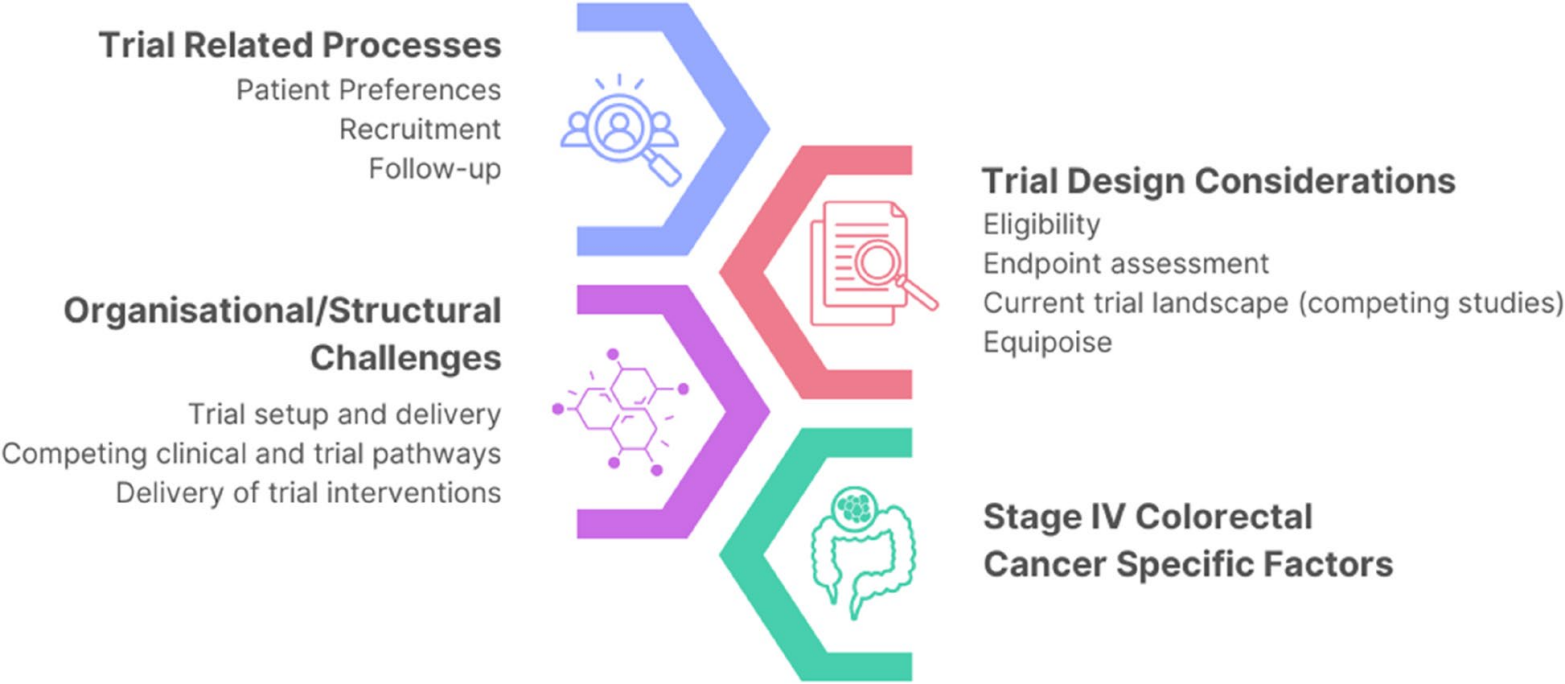
Themes and subthemes identified

Four major themes were identified, namely trial-related processes, Organisational/Structural Challenges, Trial Design Considerations, and Stage IV (Metastatic) Colorectal Cancer Specific Factors. Within each theme challenges and strategies to overcome them were identified. These were further characterised by ten subthemes illustrated in Fig. 1.

#### Trial-related processes

##### Patient preferences

Predetermined patient preferences were considered to be one of the key reasons for the difficulty in recruiting to trials. Patient opinions were formed by their own research regarding different treatments and were considered to be better informed about potential options by staff who felt this was due to the internet and social media. “There is patient preference, because they have social media, online, they are better informed of their own preferences, erm… and that’s why it is hard to include patients for randomisation.” (Chief of Principal Investigator (CI/PI) (trial closed)).

This varied for different interventions, for example, participants had encountered strong preferences for laparoscopic surgery versus open, particularly in younger patients. Clinical pathways also had an impact on this, with patients expecting a particular treatment i.e. surgery, due to being referred to a surgical specialty or specialist. Potential conflict between recruiting to randomised trials and shared decision-making processes; taking into account individual patients and their preferences, was also considered to have an impact on trial recruitment. Potential solutions included asking sites to only offer specific treatments within trial settings and not to offer these treatments to patients not participating in trials, in addition to research staff honing their approach to recruitment, offering patients a balanced view regarding treatment arms and existing evidence. “And to, have a certain story towards positions which you also… I mean, I mean, of course everyone does things in their own way, but you can give them sort of a generic story you would tell the patient as a trial team.” (Trial Co-ordinator/Manager (TC/M) (trial recruiting)). Undertaking patient and public involvement (PPI) work was also proposed to identify potential barriers to recruitment from a patient perspective.

##### Recruitment

Recruitment challenges included issues related to the recruitment encounter, such as patients being overwhelmed by the volume of information, or being shocked or surprised by the treatments proposed, unconscious bias from the clinician towards a particular intervention, difficulty communicating the complexity of the treatment options in addition to explaining the trial, and a lack of time for this process. Additionally, it was challenging for a clinician from one specialty to offer a balanced view of interventions delivered by another specialty. Other challenges included difficulty recruiting to trials where the interventions offer no direct survival benefit to the patient such as laparoscopic versus open surgery, a lack of drive to recruit at a site level due to high clinical workload, and the trial not being introduced early enough in the patient pathway, resulting in patients developing expectations regarding their treatment. “So, you would say hi to the patient and have to start from the point of view of discussing different treatments, when the patient had already been told they had been selected for liver surgery. So, that was one of the major, major fundamental problems and we did try and tackle that.” (CI/PI (trial terminated)).

Participants described solutions which they had successfully implemented within their own trials. Solutions specific to the recruitment encounter included introducing the trial early in the consultation or contacting patients prior to clinic to introduce the trial, using animated videos to explain the trial, having a concise but clear consent form, having longer clinic appointments for trial consent, involving clinicians from all involved specialties, and using only one consultant from each site to lead on consenting to a trial. Organisational approaches included screening the multi-disciplinary team (MDT) to ensure all eligible patients are identified, research nurse presence in clinic to identify participants, and combining trial visits or consent with existing appointments to reduce burden. “And our intention was to tackle that by the MDT’s identifying the patients and the patients being contacted straight away to say there is a study that might suit you rather than you are suitable for surgery.” (CI/PI (trial terminated)). Other proposed solutions which participants had implemented included the central research teams sending newsletters or pocket cards to sites to remind them to recruit, fostering competition through site recruitment leaderboards, CI visits to sites, and delivering teaching programmes regarding the running of the trial and how to present the trial to patients.

##### Follow-up

Challenges related to follow-up included logistical challenges where patients were treated across different sites, the perceived increased burden of additional follow-up appointments or the additional burden of completing quality of life questionnaires. “Sometimes when patients were referred to a regional centre for surgery, so a couple of our sites we had, you know, chemotherapy would be done at a certain place, surgery would be done at another. So, it became more difficult to get the data completed accurately, you know, by the surgical teams, for example.” TC/M (trial completed)).

Potential solutions proposed by participants included providing additional support to patients to complete follow-up, particularly whilst in hospital, asking patients to complete quality of life questionnaires in the waiting room prior to appointments, and contacting patients to remind them to complete follow-up. Having a flexible approach, conducting follow-up either in person or remotely could be beneficial, in addition to using the same follow-up schedule as standard care. The logistical challenges associated with delivering follow-up across different sites could be addressed by using paper case report forms (CRFs) that move with patient notes to facilitate data collection. Alternatively, central research teams could collect follow-up data either via post or online to reduce the burden on sites or issue regular reminders to sites to conduct follow-up visits. “So, if I ring somebody twice in the morning, I’m not gonna spend all day ringing them because I’ve got ten other people to ring. So yeah, I think getting someone else to do that is really good, online is really good.” (Research Nurse (RN) (trial completed)).

#### Organisational/structural challenges

##### Trial setup and delivery

Several challenges related to trial setup and delivery were identified including difficulty securing funding, ethical approval, local site setup, communication and language barriers in international trial settings and a lack of research infrastructure. Funding challenges included the difficulty in securing funding in the mCRC setting, and in particular for interventions with no long-term survival benefit but a possible quality of life benefit. Difficulties securing funding for surgical and interventional trials in mCRC were also encountered and presented additional challenges when delivering interventions across different specialties, centres, and Trusts. This can be further amplified in international trials from a structural and organisational perspective, in addition to translation costs. Ethical approval processes can be complex and time-consuming, particularly if multiple international approvals are required. “Erm… yeah, every, every country has their own rules, which can be completely different, their own demands. And they can also have, after they’ve done the review, they can highlight other aspects, erm, so sometimes you find yourself changing, adding on things, or changing things, when it’s already been approved by three or four ethical committees.” (TC/M (trial recruiting)). Site setup challenges included difficulty obtaining local approvals, particularly for complex interventions or where two specialties were involved. These delays in site setup subsequently impacted on the length of time available for recruitment. Communication challenges such as language barriers or time differences also impact on the time required for site setup, particularly in the international setting. A lack of research infrastructure within some participating specialties was also highlighted, in addition to challenges associated with small numbers of sites offering trial interventions.

Potential solutions to these issues described by the participants included the incoming Centralised European Ethical Review and ethical committees being receptive to feedback from doctors and patients. Streamlining ethical approval and site setup processes was also proposed in addition to highlighting the importance of having a cohesive research team with support and regular communication from the central research team. “So, we need to streamline the process to have fewer individuals that are that are doing the steps for the trial setup and if it’s being done through a trials unit, they need to have a time deadline for individuals within the trials unit to do the work, and if they are not, if they don’t have a sufficient individuals to do those bits of work that they need to do, that they need to do something about that urgently.” (CI/PI (trial terminated)).

##### Competing clinical and trial pathways

Differences between trial pathways and standard procedures also presented challenges, particularly if the delivery of the trial did not complement the structure of the healthcare system, for instance where patients were treated across different sites, which could result in difficulty collecting clinical data points, particularly for sites across different healthcare providers with different electronic systems. “So that was part of the MDT like we tried to implement that you screen at the MDT but I still think that’s a complex arrangement for patients to go through, so even when they say, yeah we’re fine, we’re ready for it, it’s a brand new process that I think is quite hard to like implement easily into normal hospital life I guess.” (TC/M (trial terminated)). Other examples included follow-up schedules for the trial being different to standard follow-up processes, requiring additional appointments. Solutions to these issues included trial teams mapping out and defining trial processes and existing patient pathways during study design and site setup with a view to ensuring cohesion. “Yeah, I think there’s and also, this is probably for a lot of trials, just mapping through the actual patient flow, at what stages would you recruit them, erm, and especially when you’ve got different treatment delivery, how that’s going to work. Like, between them, how that organisation’s going to work.” (TC/M (trial terminated)). Additionally, the potential to offer flexibility in relation to follow-up timepoints and balancing the volume of clinical data collection required against a desire to minimise the workload for sites.

##### Delivery of trial interventions

Challenges related to the delivery of trial interventions included a lack of resources and support within hospitals, including research staff shortages, particularly following the COVID-19 pandemic. Challenges specific to the delivery of certain interventions included the requirement for additional investigations or work-up, for example, lung resection which required pre-operative lung function tests. The delivery of a complex intervention or complex trial process was also identified as a factor which led to research teams disengaging from a study due to the increased workload, particularly if trial processes were felt to be onerous or diverged significantly from standard procedures at a local level. “But a lot of these things I basically do because I’m seeing this cohort of patients and out of the goodness of my heart. And so, when people start making it really difficult, it does just put you off. It’s the truth.”(RN (trial completed)). Logistical difficulties related to running trials across different specialties were also described as being a barrier to recruitment. Having co-PIs from both involved specialties could help resolve these issues, “and it’s a joint clinic with an oncologist where we sit down and rather than the half an hour for me, 45 min for them, we get an hour with two of us to go through it.” (CI/PI (trial completed)). Other solutions to challenges with the delivery of interventions included valuing and preserving experienced research teams and involving allied healthcare professionals in research to help mitigate staffing shortages across research teams. Remote consent could be helpful both in recruiting geographically distant patients and in reducing workloads for research teams.

#### Trial design considerations

##### Eligibility

The main challenge regarding eligibility related to recruitment challenges due to narrow eligibility criteria. Patients with mCRC are a heterogenous patient cohort due to patient, disease and treatment characteristics. Applying narrow windows of eligibility criteria to create a homogenous and comparable cohort reduces the overall pool of potentially eligible patients, thus further amplifying difficulties in recruitment. “And also especially in this patient group because, I mean, we’re only including patients that have synchronous colorectal liver metastases which are eligible for both the resection of the primary and the primary disease. So that, that group is already fairly small.” (TC/M (trial recruiting)). Broad eligibility criteria in this setting maybe of more value, as it potentially allows the recruitment of a larger number of patients, thus improving recruitment, “So we, I think we you know we use the screening logs to look at our entry criteria and we’d set a limit of on one of the studies so we weren’t allowing anybody over 80 to go into the study and yet we had a fit and healthy 81 year old and an 82 year old that we’d missed on our screening log. So, we went back and we changed the criteria based on the screening log.” (TC/M (trial completed)).

##### Endpoint assessment

Determining the optimal primary endpoint in mCRC during the design phase was considered to be challenging, due to the need to balance clinically relevant outcomes i.e., survival, toxicity, progression, with patient-reported outcome measures i.e., quality of life (QoL), effect size, and appropriate statistical power. Selecting the most appropriate primary endpoint was identified as a challenge, requiring an understanding of the trial interventions and patient cohort. “And, and for a trial like this, it’s nearly impossible to recruit, for example, more than 500 patients. So yeah. And then, of course your primary endpoint has to be clinically meaningful. So that’s, that’s, that’s a balance that that can be difficult during the setup phase of the trial.” (TC/M (trial recruiting)). Selecting the correct primary endpoint is essential as it dictates the overall sample size, and therefore effects recruitment and feasibility of trial delivery. It can also affect the funding of a trial or intervention, as funders and healthcare providers may consider demonstration of a survival benefit to be essential. This can be particularly challenging when considering non-curative treatments in the context of mCRC, which require different endpoints to reflect the difference in treatment intent.

Determining the most appropriate primary endpoint was felt to require an understanding of the trial interventions and effective communication and collaboration between clinicians delivering the intervention and trial statisticians and methodologists designing and powering the trial. Undertaking qualitative work, with multidisciplinary stakeholder involvement, was identified as a potential strategy to ensure the optimal primary endpoint is selected for mCRC trials, whilst considering patient acceptability and trial deliverability. Erm, and again, in a surgical trial now, I would always try to push the importance of having an upfront qualitative study, not just about the practicalities of recruiting, but also about… So like for example, we’ve had a qualitative study where we’ve tried to engage with all the surgeons and tried to get them talking about how they perform the operation, how they do the preoperative prep and stuff, and understand how heterogeneous it is, and hone in on what we need to be collecting during the trial to capture all of that.” (Statistician (trial terminated)).

##### Current trial landscape (competing studies)

Participants described challenges recruiting participants to trials due to competing studies and the overall small cohort of potentially eligible patients, “Is that you can also have like a sort of competition between specialties. I mean, competition is not maybe the right word, but competition between specialties and recruiting patients for trials.” (TC/M (trial recruiting)). Multiple competing trials recruiting at the same time in mCRC can lead to variable recruitment trajectories based on individual centres’ priorities and patient preferences.

Strategies identified to overcome this included reviewing the current landscape to identify open and recruiting trials and engaging in collaborative discussions both with key stakeholders and trial management groups of other clinical trials prior to designing and opening new trials in this setting. “So, we spent quite a lot of time in the early trial stages getting opinions on the trial format and anything that might inhibit trial recruitment. And that information was obtained from surgeons, from oncologists, from patients and from trial nurse specialists. So, everybody that’s involved in patient management. And the consensus was that the trial was feasible and that the question was important.” (CI/PI (trial terminated)). Designing trials to either co-enrol, share ethics protocols and data collection platforms, or follow up protocols could also help improve the efficiency of trial design and encourage recruitment in mCRC.

##### Equipoise

Equipoise was identified as an important consideration for trial design, with several related challenges identified from a clinician perspective, particularly in relation for preferences for treatment arms causing difficulties with recruitment and randomisation. This included clinician preferences being informed by their own experiences and not wanting to enrol patients due to their preference for one treatment arm. “And then you get the awful thing of them saying, well, why don’t you randomize them and see what they get? And you have to sort of explain, that’s really all the point of the trial and that sort of thing! (laughter).” (RN (trial completed)). Difficulties aligning views of clinicians from different specialties were also described, with surgeons often preferring a surgical treatment arm over radiological or medical alternatives. This could lead to clinicians not recruiting to a trial or presenting trial interventions to patients in an unbalanced way.

Undertaking pre-trial qualitative work specifically to address issues of equipoise is of huge benefit in developing trial and intervention-specific training bundles to minimise the impact of lack of equipoise. Proposed solutions to overcome challenges related to equipoise included developing an understanding of current evidence, community equipoise and clinician-specific training. Identifying lead clinicians for each centre who could act as senior clinical champions for a trial and gaining endorsement of a trial from relevant surgical organisations can help with addressing clinician and community equipoise. “So, we had clinicians that put their hands up from every single unit and said, “Yes, we think that there is equipoise and we’re happy to recruit into this trial”. And what I did from that point of view was that I said well, that’s good that you feel that there’s equipoise, could you discuss that with all the other clinicians in your group and make sure that there is consensus and that everybody is happy to recruit patients into this trial because I was worried that, Okay it’s like saying well as anybody interested in this trial? And people that are enthusiastic put their hands up but behind them there’s fifty people that don’t want to see the trial working.” (CI/PI (trial terminated)).

#### Stage IV colorectal cancer-specific factors

Unique issues were identified in relation to undertaking trials in mCRC, these included disease progression, treatment complexity, and disease-related complications. Disease progression in mCRC is unpredictable and can lead to attrition due to patients dropping out or being lost to follow-up. “But sadly, some of them will progress and obviously, you know, then this can be patients here undergoing palliative chemotherapy and things like that. So, it’s also, you know, find the right balance between what’s right for the study, but also was right for the patient.” (RN (trial completed)). This can particularly impact on the collection of long-term QoL data.

Participants also highlighted the complexity of the management of mCRC as a major consideration for trial design; the complexity of the management of mCRC meaning it can be difficult to establish the effect of an intervention alongside the multiple other interventions and treatments patients have received throughout the course of their treatment. Furthermore, these treatments are often delivered by several clinicians i.e. colorectal surgeons, hepatobiliary surgeons and oncologists, across a range of hospital departments and sites. This can make it difficult to seamlessly embed trial processes within routine clinical care. The impact of emergency treatment on trial processes was also identified, as disease-related complications can lead to the need for patients requiring emergency surgical or radiological interventions, which may lead to patients dropping out of the trial, or not being approached for participation. “So the colorectal cancer sometimes has symptoms, like intestinal obstruction, so in our study, erm, the patients with intestinal obstruction, the patients had to undergo surgery before entering our study … so we cannot start the treatment for the liver metastases” (RN (trial completed)). In relation to addressing these challenges, ensuring patients requiring emergency treatment are appropriately reflected with the trial schema and design is important to ensure the generalisability of future results, determine the safety profile of different treatment profiles, and determine attrition rates in this complex cohort to inform future sample size calculations.

## Discussion

Conducting clinical trials in mCRC is complex due to a range of clinical, patient and trial-related factors. We identified four key themes which underpinned the difficulties in trial conduct in this setting: Trial Related Processes, Organisational/Structural Challenges, Trial Design Considerations, and Stage IV Colorectal Cancer Specific Factors. These themes reflect the broad-based challenges facing researchers and clinicians conducting RCTs in the setting of mCRC, which appear amplified when embedding complex interventions i.e. surgery within complex clinical settings. The main challenge in conducting trials in mCRC is the difficulty in creating cohesion between existing clinical pathways and trial processes, with the timely approach of patients, balancing complex treatment decisions against the backdrop of trial recruitment.

Recently there have been positive developments regarding trials in this setting, such as the successful recruitment to the TransMet trial regarding liver transplantation for mCRC [25]. However, overall participation of patients with CRC in interventional trials is relatively low, with a rate of around 5% reported for individuals diagnosed with CRC in England [26]. There are numerous potential benefits to be gained from increasing participation in clinical trials, such as improving treatment outcomes and access to existing treatments. Improved survival outcomes for patients with CRC have also been associated with receiving treatment in hospitals with higher participation in interventional trials [26], as such, better engagement with clinical trials may benefit patients overall. Improving participation in clinical trials should be a priority for policy makers, clinicians, and patients, with a view to improving outcomes. However, the challenges associated with delivering trials in this setting pose significant barriers. Previous studies in similar disease settings have reported challenges including access to trials, insufficient time or resources, and complexity in trial procedures [27–29]. Potential strategies to overcome these challenges which have previously been described include maintaining high levels of engagement from the CI and TMG, decentralising some trial activities, utilising digital healthcare technologies, and considering pragmatic clinical trial design [27, 28, 30]. The results of this study support and build on these findings, focusing more specifically on patients with mCRC and on trials including surgical interventions. The study findings reiterate the complexity of both the management of mCRC and delivering trials in this context, with important implications for researchers to consider for future trial development and delivery. The findings strongly support the early involvement of stakeholders through both qualitative work and PPI, and close working practices with MDTs given the central role of the MDT in the management of this complex patient group. These changes, alongside measures such as delivering staff training regarding equipoise, and changes to study design and delivery, such as centralising trial follow-up, could be implemented and assessed utilising the Quintet Recruitment Intervention (QRI) [31] in future studies. Initiatives including the GeneRAtiNg StUdent Recruiters for Surgical TriaLs (GRANULE) course have shown the potential success of delivering targeted training [24]. International collaboration in the mCRC setting is also of huge importance, as it increases the potential pool of eligible patients and allows for generalisability across a range of populations.

Significant strengths of the study include recruiting research team members across a range of roles, which included CIs, study co-ordinators, trial managers, research nurses and a statistician. Our work focused on key aspects of trial design and delivery and was not limited to recruitment challenges only, thus providing a broader view on the challenges and available strategies for triallists in this arena. One of the major limitations of the study is the small number of participants representing a limited selection of the eligible trials identified, with only one trial-related researcher interviewed for some of the trials, which may have presented a unidimensional view. There were some difficulties encountered in contacting and interviewing all trial team members, in particular with regards to contacting specialist research nurses and for trials which had either completed or closed. Participants were approached via email; it is possible that some of those approached may have changed roles or their current workplace and therefore may have not received the email invitation. The six trials represented related predominately to interventions for liver metastases and therefore broader issues related to specific clinical settings i.e. multiorgan metastatic disease or interventions i.e. biomarker specific treatment regimens may not have been reported. Additionally, these trials focused on interventions with curative intent, and trials of palliative interventions may present different challenges, such as the selection of primary endpoints, as highlighted. Despite the small number of trials included, the study was felt to have sufficient information power [23], with key informants and a rich dataset. Furthermore, interviews were only undertaken in English, which could have been a barrier to participation for some trial teams and may have excluded international trial teams from participating. The interviewer’s background may have also influenced the study results, given they have experience working in challenging recruitment settings. Only one of the trials included in this study considered biological subtypes in their eligibility criteria. Heterogeneity in relation to tumour biology should be a significant consideration for future trials and may further compound some of the recruitment challenges described by further narrowing eligibility criteria. Treatment for mCRC is increasingly considered by subtype rather than as one entity, reflecting the significant impact of tumour biology on both prognosis and treatment options, such as immunotherapy for patients with high microsatellite instability (MSI-H) [32, 33]. This development was evident in the TransMet trial, where patients with BRAF tumour mutation were excluded due to its associated poor prognosis [25]. Further work may be required to identify the impact this will have on recruitment to mCRC trials.

## Conclusions

Overall, this study provides valuable insights regarding the challenges associated with conducting RCTs in mCRC alongside stratified approaches to addressing these challenges in future trials, from the design stage through to delivery. Despite the significant complexity associated with conducting trials in this setting, many of the solutions proposed include simple measures which could be easily implemented. Further work to evaluate the effectiveness of these proposed solutions would be beneficial and could be undertaken using the QRI. Crucially, provision of funding, combined with high levels of engagement and collaboration, both within the colorectal community and across all involved specialties and stakeholders, will be central to the successful delivery of future trials in mCRC.

## Supplementary Information

Additional File 1. Topic Guide for Interviews.

Additional File 2. Table 1: Subcategories and Characteristics of the Trials Identified.

## Data Availability

The datasets generated and analysed during the current study are not publicly available. However, may be available from the corresponding author on reasonable request.

## Abbreviations

CI: Chief investigator
CRC: Colorectal cancer
CRF: Case report form
EU: European Union
GRANULE: GeneRAtiNg StUdent Recruiters for Surgical TriaLs
mCRC: Metastatic colorectal cancer
MDT: Multi-disciplinary team
NIH: National Institutes of Health
PI: Principal investigator
PPI: Patient and public involvement
QoL: Quality of life
QRI: Quintet Recruitment Intervention
RCT: Randomised controlled trial
RFA: Radiofrequency ablation
RN: Research nurse
SRQR: Standards for Reporting Qualitative Research
TC/M: Trial co-ordinator/manager
TMG: Trial management group
WHO: World Health Organisation
