# Supplementary material for: A qualitative study to understand the challenges of conducting randomised controlled trials of complex interventions in metastatic colorectal cancer
Source: Trials. 2025 Mar 19;26:98. doi: 10.1186/s13063-025-08811-z (PMC11924622; doi:10.1186/s13063-025-08811-z)
Supplement: Supplementary file 1 — Additional File 1. Topic Guide for Interviews. [file 13063_2025_8811_MOESM1_ESM.pdf]

UNIVERSITY OF LEEDS

ctru

Topic Guide for Interviews

Understanding the challenges of conducting trials in metastatic colorectal cancer

1. Introduction

- Study overview
- Funder – Bowel Diseases Research Foundation
- Outline selection of participants

2. Aims of Study

- Informal discussion to gauge challenges to conducting trials in metastatic colorectal cancer

3. Confidentiality

- Audio-recording of all interviews
- Reiterate anonymity of all data
- Ensure obtained consent
- Voluntary withdrawal at any time during the interview

4. Audio-recorded Interview

a. Opening Questions

- Overview of trial
- Role within the trial or trial management group

b. Questions

- Can you tell me of any specific challenges you think are unique to conducting a trial in patients with metastatic colorectal cancer?
- Have you experienced any challenges in conducting a trial in metastatic colorectal cancer? What are these?
- What do you think the challenges are in recruiting patients with metastatic colorectal cancer to clinical trials?
- What do you think the challenges are in following up patients with metastatic disease?
- Have you employed any strategies to help overcome any recruitment/follow up challenges you may have faced?

5. Close of interview
