# Supplementary material for: A qualitative study to understand the challenges of conducting randomised controlled trials of complex interventions in metastatic colorectal cancer
Source: Trials. 2025 Mar 19;26:98. doi: 10.1186/s13063-025-08811-z (PMC11924622; doi:10.1186/s13063-025-08811-z)
Supplement: Supplementary file 2 — Additional File 2. Table 1: Subcategories and Characteristics of the Trials Identified. [file 13063_2025_8811_MOESM2_ESM.pdf]

Table 1: Subcategories and Characteristics of the Trials Identified

Registry	Trial Registry Identifier	Country	Trial Name	Institute	Year	Sample Size (if listed)	Interventions	Primary Endpoint	Current Status
**Surgical resection of the primary tumour**									
WHO	ChiCTR 2000035397	China	The survival value of primary tumour resection in metastatic colorectal cancer based on tumour biological behaviour	The First Affiliated Hospital of Tsinghua University	2020	446	Chemotherapy plus surgery<br>Chemotherapy alone	Progression-free survival	Recruiting
Clinical trials, WHO	NCT 01606098	Netherlands	CAIRO4 – The Role of Surgery of the Primary Tumour in Patients with Synchronous Unresectable Metastases of Colorectal Cancer	Radboud University Medical Centre Nijmegen	2012		Surgery followed by chemotherapy<br>Chemotherapy	Overall survival	Completed
ISRCTN	ISRCTN 30964555	Germany	Influence of primary tumour resection on the course of disease in patients with metastatic colon cancer and unresectable metastases	University Hospital Carl Gustav Carus Dresden	2011	392	Surgical resection of the tumour prior to chemotherapy<br>Chemotherapy without surgical resection	Overall survival	Completed

Clinical trials	NCT 01978249	South Korea	The role of primary tumour resection in colorectal cancer patients with asymptomatic, synchronous unresectable metastasis	Yonsei University	2013	52	Primary tumour resection followed by chemotherapy	Overall survival	Completed
							Chemotherapy first without surgical resection		
WHO, UMIN	UMIN 000008147	Japan	iPACS - RCT comparing primary tumour resection plus chemotherapy with chemotherapy alone in incurable stage IV colorectal cancer: JCOG1007	National Cancer Center Hospital, Tokyo	2012	770	Primary resection followed by chemotherapy	Overall survival	Completed
							Chemotherapy alone		
WHO	ChiCTR-TRC-13003307	China	The survival benefit of preoperative chemotherapy before primary tumour resection on synchronous metastatic colorectal cancer	West China Hospital of Sichuan University	2017	189	Pre-operative chemotherapy followed by surgical resection of the primary tumour / Surgical resection of the primary tumour followed by chemotherapy	Overall survival	Completed
							Chemotherapy alone		
Clinical trials, WHO, EudraCT	2013-001688-22 and NCT 02015923	Spain	Randomized multicentre trial in colorectal cancer patients with non-	Servicio de Cirugía General y Digestiva	2013	107	Colonic resection followed by chemotherapy	Overall survival	Recruiting

			resectable metastasis. Impact of tumour resection versus chemotherapy alone in survival	Hospital Universitario de Bellvitge			Chemotherapy alone		
Clinical trials	NCT 01086618	UK	Chemotherapy With or Without Surgery in Treating Patients with Metastatic Colorectal Cancer That Cannot Be Removed by Surgery	University College London Hospitals	2010	500	Surgery and chemotherapy	Overall survival	Completed
							Chemotherapy		
Clinical trials, WHO	NCT 02149784	China	Effectiveness Study of Resection of Primary Tumour in Stage IV Colorectal Cancer Patients	Sun Yat-sen University	2014	480	Surgical resection for patients who respond to chemotherapy	Overall survival	Recruiting
							Chemotherapy		
**Comparing different management strategies for the primary tumour**									
Clinical trials	NCT 01157806	Poland	Palliative radiotherapy and chemotherapy instead of surgery in symptomatic rectal cancer with synchronous unresectable metastases: a phase II study	Maria Sklodowska-Curie National Research Institute of Oncology	2010	20	Radio-chemotherapy instead of surgery	Percentage of patients not requiring palliative surgery	Completed
							Standard surgery		

WHO, UMIN	UMIN 000009715	Japan	ENCORE - RCT Comparing Laparoscopic Surgery with Open Surgery in Palliative Resection of Primary Tumour in Incurable Stage IV Colorectal Cancer: Japan Clinical Oncology Group Study JCOG 1107	Oita University Faculty of Medicine	2007	194	Laparoscopic primary tumour resection followed by chemotherapy<br>Open primary tumour resection followed by chemotherapy	Progression-free survival	Recruiting
Clinical trials, WHO	NCT 03451643	Italy	Stenting and Resection for Stage IV Colorectal Cancer Endoscopic Stenting and Colorectal Resection in Stage IV	University of Roma La Sapienza	2018	50	Endoscopic stent and chemotherapy<br>Colorectal resection and chemotherapy	60-day morbidity and mortality, overall survival	Completed
**Metastasectomy versus chemotherapy/other therapies**									
Clinical trials	*NCT 01106261*	*UK*	*PulMiCC – A randomised trial of Pulmonary Metastasectomy in Colorectal Cancer*	*University College London*	*2012*	*N/A*	*Pulmonary metastasectomy*<br>*Active monitoring*	*Feasibility of recruitment*	*Completed*
Clinical trials	NCT 01545141	USA	Chemokine-Modulatory Regimen for Recurrent Resectable Colorectal Cancer	UPMC Hillman Cancer Center, Pittsburgh	2012		Chemokin modulatory regimen prior to surgery (5 MU/m2) / Chemokin modulatory regimen prior to surgery (10 MU/m2) /	Change in the Number of Tumour-infiltrating CD8+ Cells	Terminated

							Chemokin modulatory regimen prior to surgery (20 MU/m2)		
							Surgical resection		
Clinical trials	NCT 03373188	USA	VX15/2503 and Immunotherapy in Resectable Pancreatic and Colorectal Cancer	Emory University	2017		VX15/2503, surgery, VX15/2503, ipilimumab, surgery, VX15/2503, nivolumab, surgery	Tumour cluster of differentiation 8+ (CD8+) T cell infiltration	Unknown
							Surgical resection		
Clinical trials, WHO	NCT 04912258	China	Preoperative Irinotecan Drug-eluting Beads for Patients with Unresectable Colorectal Liver Metastasis	Fudan University, Shanghai	2021	80	DEB-TACE before liver surgery	Liver relapse-free survival	Unknown
							Direct liver surgery		
Clinical trials, WHO	NCT 01856322	USA	Surgery Plus Sulindac or Surgery Alone for Advanced Colorectal Cancer	National Cancer Institute	2013		Metastasectomy plus Sulindac	Difference in circulating S100A4 transcript levels	Terminated
							Metastasectomy alone		
WHO	NTR4893 and 2013-004952-39	Netherlands	CHARISMA - Neo-adjuvant chemotherapy followed by surgery	Erasmus MC Cancer Institute	2014	224	Chemotherapy followed by surgical resection	Overall survival	Pending

			versus surgery alone in high-risk patients with resectable colorectal liver metastases				Surgical resection		
Clinical trials, WHO	NCT 04840186	Norway	EXCALIBUR3 - EXtended CriteriA Treatment for Liver Metastases and Heavy Tumour BURden	Oslo University Hospital	2021	62	Chemotherapy and liver resection	Overall survival	Recruiting
							Chemotherapy		
Clinical trials, WHO	NCT 02738606	USA	Liver Surgery and Chemotherapy in Treating Patients With Colorectal Cancer with Liver Metastases That Can Be Removed by Surgery and Lung Metastases That Cannot Be Removed by Surgery	MD Anderson Cancer Center	2016	80	Patients undergo hepatectomy and receive chemotherapy at the discretion of treating oncologist. Patients whose lung tumours become able to be removed by surgery with chemotherapy may undergo lung metastasectomy	Overall survival	Recruiting
							Patients receive chemotherapy at the discretion of the treating oncologist. Patients whose lung tumours become able to be removed by surgery with chemotherapy may undergo lung metastasectomy		

Clinical trials	NCT 03599752	USA	Chemotherapy and/or Metastasectomy in Treating Patients with Metastatic Colorectal Adenocarcinoma with Lung Metastases	MD Anderson Cancer Center	2018	365	1A. Low risk patients receive standard of care chemotherapy for 3 months prior to and 3 months after undergoing metastasectomy<br>1B. Low risk patients undergo metastasectomy<br>2A. High risk patients undergo metastasectomy<br>2B. High risk patients continue standard of care chemotherapy for 6 months in the absence of disease progression or unacceptable toxicity. Patients with stable disease or radiographic response after 6 months may then cross over to Group 2A.<br>Chemotherapy	Recurrence free survival and overall survival	Recruiting
**Comparing multiple therapies (Surgery, SBRT, Ablation) to chemotherapy alone**									
Clinical trials, WHO	NCT 05673148	USA	ERASur - Testing the Addition of Total Ablative Therapy to	National Cancer Institute	2023	364	Patients undergo total ablative therapy, consisting of SABR with or	Overall survival	Unknown

			Usual Systemic Therapy Treatment for Limited Metastatic Colorectal Cancer				without surgical resection and/or microwave ablation		
							Chemotherapy		
ISRCTN, Clinical trials, WHO	*ISRCTN 15067672 NCT 01792934 and EUCTR 2011-005003 -32-NL*	*Netherlands*	*A randomised multicentre clinical trial for patients with multi-organ, colorectal cancer metastases comparing the combination of chemotherapy and removing as many visible tumours as possible by surgery or other means versus chemotherapy alone*	*Radboud University Medical Centre Nijmegen*	*2020*	*478*	*Chemotherapy and maximal tumour debulking including surgery, RFA, transarterial chemoembolization*	*Overall survival*	*No longer recruiting*
							Chemotherapy		
Clinical trials	NCT 05062720	USA	Local Consolidative Therapy in Colorectal Cancer	Fox Chase Cancer Center	2021		Local Consolidative Therapy (LCT) defined as surgical resection or stereotactic body radiotherapy (SBRT) plus systemic therapy	Progression-free survival	Terminated
							Systemic therapy alone		
**Timing of chemotherapy and liver resection**									

WHO	*JPRN-UMIN 000007787*	*Japan*	*EXPERT – Randomised phase III trial of surgery followed by mFOLFOX6 as adjuvant chemotherapy versus peri-operative mFOLFOX6 plus cetuximab for KRAS wild type resectable liver metastases of colorectal cancer*	*The University of Tokyo*	*2012*	*N/A*	*Pre-operative chemotherapy (mFOLFOX6 plus cetuximab) 6 cycles followed by post-operative chemotherapy (mFOLFOX6 plus cetuximab)*	*Progression free survival*	*Completed*
							Surgical hepatectomy followed by chemotherapy (mFOLFOX6)		
ISRCTN, WHO	ISRCTN 32401805	UK	Investigating the optimal scheduling of chemotherapy in patients with colorectal and liver cancer	Clinical Trials Unit Southampton	2011	78	12 weeks of standard care chemotherapy pre-operatively, surgery to resect colorectal liver metastases, then 12 weeks of standard care chemotherapy post-operatively	Feasibility of the trial to proceed to phase II	Terminated
							Surgery to resect colorectal liver metastases followed by 24 weeks standard chemotherapy		
Clinical trials	NCT 01189227	USA	Combination Chemotherapy Before or After Surgery in Treating	NSABP Foundation Inc	2010	9	Perioperative chemotherapy and hepatic resection	Recurrence free survival	Terminated

			Patients with Colorectal Cancer with Liver Metastases That Could Be Removed by Surgery				Hepatic resection and postoperative chemotherapy		
**Ablative therapies versus liver resection**									
ISRCTN	*ISRCTN 52040363*	*UK*	*LAVA – Liver resection surgery versus thermal Ablation for colorectal liVer metAstases*	*University of Leeds*	*2016*	*330*	*Thermal ablation of hepatic metastases*<br>*Surgical resection*	*2-year disease-free survival*	*Terminated*
Clinical trials, WHO	NCT 02866344	USA	Resection Versus Microwave Ablation for Resectable Colorectal Cancer Liver Metastases	Carolinas Medical Center	2016		Microwave ablation<br>Hepatic resection	Local disease control	Terminated
Clinical trials, WHO	NCT 05129787	Norway	NEW-COMET – Ablation vs Resection of Colorectal Cancer Liver Metastases	Oslo University Hospital	2021	230	Thermal ablation of hepatic metastases<br>Liver resection	Local tumour progression	Recruiting
Clinical trials, WHO	NCT 03088150	Netherlands	COLLISION - Colorectal Liver Metastases: Surgery vs Thermal Ablation	VU University Medical Center, Amsterdam	2017	618	Thermal ablation of hepatic metastases<br>Resection of hepatic metastases	Overall survival	Recruiting

**Comparing surgical approaches to manage liver metastases**									
WHO	NCT 02758977	Switzerland	ALPPSforCRLM - Associating Liver Partition with Portal Vein Ligation for Staged Hepatectomy (ALPPS) vs. Two-Stage Hepatectomy for Marginally Resectable Colorectal Liver Metastases (CRLM)	University of Zurich	2016	214	ALPPS	One year disease-free survival	Unknown
							Two Stage Hepatectomy		
WHO	ChiCTR 2000035844	China	Clinical study of total laparoscopic surgery and microwave ablation in patients with colorectal cancer with liver metastases	Ruijin Hospital North, Shanghai	2020	80	Laparoscopic radical treatment of colorectal cancer and microwave ablation of liver metastases	Early severe complication and operative mortality	Unknown
							Open radical treatment of colorectal cancer and liver metastasis resection		
Clinical trials	NCT 04798898	Denmark	ISCOLIM - Improving Survival of Colorectal Liver Metastases by RFA-mediated Immunostimulation	Rigshospitalet, Denmark	2021	200	Preoperative partial RFA necrosis in the liver metastasis followed by liver resection	Overall survival	Recruiting
							Liver resection		
Clinical trials	NCT 01516710	Norway	Oslo-CoMet - Oslo Randomized Laparoscopic Versus	Oslo University Hospital	2012	280	Laparoscopic liver resection	Perioperative morbidity	Completed

			Open Liver Resection for Colorectal Metastases Study				Open liver resection		
Clinical trials, WHO	NCT 03131778	Belgium	The Inflammatory Response to Stress and Angiogenesis in Liver Resection	University Hospital, Ghent	2017	40	Pure laparoscopic liver resection without hand assistance	Differences in inflammatory response	Completed
							Conventional open liver resection through subcostal incision		
Clinical trials, WHO	NCT 02215577	Sweden	LIGRO - ALPPS Versus Portal Vein Embolization (PVE)	Linkoping University Hospital	2014	100	In-situ liver split at time when portal vein ligature is performed (ALPPS)	Surgical success rate, the rate of liver resection in each study arm	Completed
							In-situ liver split at time when portal vein ligature is performed		
Clinical trials	NCT 01479608	Norway	Liver Transplantation and Colorectal Cancer	Oslo University Hospital	2011	25	A. Liver transplantation<br>B. Liver transplantation for non-resectable metanchronous disease<br>C. Liver transplantation for non-resectable synchronous disease<br>D. Liver transplantation for non-resectable synchronous disease	Overall survival	Recruiting
							Surgical resection		

Clinical trials, WHO	NCT 02642978	China	Robot-assisted Procedure Versus Open Simultaneous Resection of Colorectal Cancer with Liver Metastases	Fudan University, Shanghai	2015	160	Robot-assisted, simultaneous radical resection of both colorectal cancer and liver metastasis	30-day complication	Recruiting
							Traditional open simultaneous radical resection of both colorectal cancer and liver metastasis		
Clinical trials	NCT 02350166	China	Short-term Effects of LASI Surgery Versus Conventional Laparotomy for Colorectal Liver Metastasis	West China Hospital	2015	40	Laparoscopic surgery or laparoscopic-assisted small-incision for resection of laparoscopic colorectal tumour combined with synchronously small-incision open resection of liver metastasis	30-day complication	Unknown
							Conventional laparotomy for simultaneously resection of both primary colorectal tumour and liver metastasis		
Clinical trials, WHO	NCT 03895723	China	Minimally Invasive Versus Open Liver Resection for Patients with Colorectal Cancer Liver Metastases	Fudan University, Shanghai	2019	220	Minimally Invasive procedure contains laparoscopic and robotic liver resection	Disease-free survival	Recruiting
							Traditional open surgery for liver resection		

Clinical trials, WHO	NCT 01073358	Germany	RELY - Resection of Colorectal Liver Metastases with or Without Routine Hilar Lymphadenectomy	University Hospital Carl Gustav Carus Dresden	2010	166	Hilar lymphadenectomy is performed before actual resection of the colorectal liver metastases<br>Resection of colorectal liver metastases without routine hilar lymphadenectomy	Rate of disease recurrence	Recruiting
Clinical trials, WHO	NCT 04678583	Germany	ARMANI - Anatomical Resection of Liver MetAstases iN patIents with RAS-mutated Colorectal Cancer	University Hospital Carl Gustav Carus Dresden	2020	240	Removal of the entire, tumour-bearing liver segment(s)<br>Metastasectomy with a margin of healthy liver tissue irrespective of segmental borders	Intrahepatic disease-free survival	Recruiting
WHO	NCT 01441856	Netherlands	The ORANGE II PLUS - Trial: Open Versus Laparoscopic Hemihepatectomy	Maastricht University	2011	350	Open or laparoscopic left hemihepatectomy<br>Open or laparoscopic right hemihepatectomy	Time to functional recovery	Completed
WHO	*NCT 00874224*	*Netherlands*	*ORANGE II – Surgical recovery after left lateral hepatic sectionectomy: laparoscopic versus open surgery*	*Maastricht University Medical Center*	*2009*	*110*	*Open left lateral hepatic sectionectomy*<br>*Laparoscopic left lateral hepatic sectionectomy*	*Time to functional recovery*	*Completed*
**Comparing surgical approaches to manage lung metastases**									
Clinical trials	NCT 03113318	Denmark	Mediastinal Lymph Node Dissection in Conjunction With	Rigshospitalet, Denmark	2017	200	Lymph node dissection and pulmonary metastasectomy	Overall survival	Recruiting

			Pulmonary Metastasectomy From Colorectal Cancer				Pulmonary metastasectomy only		
**Timing of resection for primary cancer versus liver metastases**									
Clinical trials	*NCT 05138094*	*Italy*	*LIVACOR – Minimally invasive LIVer and simultaneous COlorectal Resection*	*Fondazione Poliambulanza Istituto Ospedaliero*	*2021*	*82*	*Minimally invasive resection of both primary colorectal carcinoma and liver metastases in one procedure*	*Time to functional recovery*	*Recruiting*
							Minimally invasive resection of the primary colorectal carcinoma and liver metastases in two stages		
Clinical trials	NCT 01056809	Sweden	Treatment Strategies for Primarily Generalized Colorectal Cancer	Department of Surgery in Östergötland	2010	15	First treatment of metastases with chemotherapy and if possible surgery, later resection of primary colorectal tumour if hope for cure or if symptoms develop that necessitate treatment	Overall survival	Terminated
							First resection of the primary colorectal tumour, then treatment of metastases with		

							chemotherapy and if possible surgery		
ISRCTN, WHO	ISRCTN 46692480	UK	RVERS - Systemic chemotheRapy and the liVEr-fiRSt approach compared to index colorectal resection for colorectal cancer presenting with synchronous liver metastases	Manchester Royal Infirmary	2012	120	Systemic chemotherapy, liver resection, adjuvant chemotherapy followed by colorectal resection	Disease-free survival	Terminated
							Bowel cancer surgery first, neoadjuvant chemotherapy and liver resection		

Trials in italic text are represented by the study participants
